# Menstrual disturbances in British Servicewomen: A cross-sectional observational study of prevalence and risk factors

**DOI:** 10.3389/fnut.2022.984541

**Published:** 2022-10-19

**Authors:** Thomas J. O'Leary, Caitlin Perrett, Charlotte V. Coombs, Rebecca L. Double, Nicky Keay, Sophie L. Wardle, Julie P. Greeves

**Affiliations:** ^1^Army Health and Performance Research, Army Headquarters, Andover, United Kingdom; ^2^UCL Division of Surgery and Interventional Science, London, United Kingdom; ^3^UCL Division of Medicine, London, United Kingdom; ^4^Norwich Medical School, University of East Anglia, Norwich, United Kingdom

**Keywords:** amenorrhoea, eating disorder, low energy availability, Female Athlete Triad, military

## Abstract

Female athletes are at increased risk of menstrual disturbances. The prevalence of menstrual disturbances in British Servicewomen and the associated risk factors is unknown. All women under 45 years in the UK Armed Forces were invited to complete a survey about demographics, menstrual function, eating and exercise behaviors, and psychological well-being. 3,022 women participated; 18% had oligomenorrhoea or amenorrhoea in the last 12 months, 25% had a history of amenorrhoea, and 14% had delayed menarche. Women who sleep ≥ 8 h were at a lower risk of a history of amenorrhoea than women who sleep ≤ 5 h [odds ratio (95% confidence intervals) = 0.65 (0.48, 0.89), *p* = 0.006]. Women who completed > 10 days of field exercise in the last 12 months were at higher risk of a history of amenorrhoea than women completing no field exercise [1.45 (1.13, 1.85), *p* = 0.004]. Women at high risk of an eating disorder (FAST score >94) were at higher risk of oligomenorrhoea or amenorrhoea [1.97 (1.26, 3.04), *p* = 0.002] and history of amenorrhoea [2.14 (1.63, 2.79), *p* < 0.001]. Women with symptoms of anxiety or depression were at higher risk of a history of amenorrhoea [1.46 (1.20, 1.77) and 1.48 (1.22, 1.79), *p* < 0.001]. British Servicewomen had a similar prevalence of menstrual disturbances to some endurance athletes. Eating disorders, sleep behaviors, and management of mental health, provide targets for protecting health of the reproductive axis.

## Introduction

Female athletes are at risk of menstrual disturbances as a result of low energy availability, described in the Female Athlete Triad (Triad) (1, 2) and Relative Energy Deficiency in Sports (RED-S) (3, 4) frameworks. Amenorrhoea is the absence of menses (> 90 days between cycles) and oligomenorrhoea is too few menses (> 35 days between cycles) and can be caused by suppression of the hypothalamic pituitary ovarian (HPO) axis (1, 5). The Triad reflects the coexistence of low energy availability, menstrual disturbances, and low bone mineral density (1, 2). The RED-S syndrome expands the Triad to include other health and performance outcomes affected by low energy availability (3, 4). Disordered eating or the desire to be lean are pre-dispositions for the Triad and RED-S, but other risk factors such as unavoidable high exercise volumes, unintentional undereating, activity type, stress, and psychological ill-health likely contribute to menstrual disturbances (1–3). Better understanding the prevalence of menstrual disturbances and associated risk factors is important for managing health and performance in Servicewomen.

Military roles can be physically and psychologically demanding, and energy intake may not always match high total energy expenditures (6). High exercise volumes with insufficient energy intake, psychological stress, and poor sleep are all potential risk factors for disturbed menstrual function in Servicewomen [reviewed in (5, 6)]. Metabolic and endocrine studies in military personnel report acute (up to 8 weeks) responses to training characteristic of low energy availability, mainly in men (6). There are fewer data in Servicewomen, but women may be more sensitive to the metabolic disturbances induced by low energy availability (6), and are at increased risk (~3-fold) of bone stress injuries during military training (7) compared with men. High energy expenditures and low energy availability (8), disrupted HPO function and menstrual disturbances (9), psychological stress and poor sleep with normal hypothalamic pituitary adrenal (HPA) axis function (10), and bone stress injuries (11) have been reported in women undergoing British Army basic training. Basic military trains civilians to be soldiers, typically involves physical training (e.g., load carriage, running, resistance training) and learning military skills (e.g., combat field exercises, drill), and is one of the most arduous parts of a military career (12, 13). Whilst these experimental data in basic training show risk factors for menstrual disturbances and associated endocrine changes, there are no data examining the prevalence of menstrual disturbances and associated risk factors in women in the entire UK Armed Forces. Typically, disturbances to endocrine function and the menstrual cycle have been attributed to low energy availability, but Servicewomen are exposed to other stressors including poor sleep and psychological stress that could also influence menstrual function.

The primary aim of this study was to determine the prevalence of menstrual disturbances in British Servicewomen. Primary outcomes were prevalence of oligomenorrhoea or amenorrhoea, history of amenorrhoea, and delayed menarche. Secondary aims were to identify the risk factors for oligomenorrhoea or amenorrhoea and history of amenorrhoea: demographics, job role, exercise volume, risk of eating disorders, sleep, and psychological well-being were also measured. The susceptibility of women to menstrual disturbances in response to behavioral and environmental factors can differ year on year (14) and so we explored risk factors for current and history of menstrual disturbances. Better understanding menstrual disturbances and associated risk factors will help identify if outcomes associated with the Triad and RED-S are clinical risks in military employment.

## Materials and methods

### Participants and study design

All women aged under 45 years in the UK Armed Forces (both Regular and Reserves) were invited to take part in this observational trial and complete an online questionnaire by email. The questionnaire was hosted electronically from July to December 2021. To target those without regular access to email, the questionnaire was also advertised through routine distribution of military orders, on posters around military barracks, and on social media networks. Participants were compensated with a £5 voucher. The exclusion criteria were: aged 45 or older; polycystic ovary syndrome, or; currently taking any medications (other than hormonal contraceptives) that affect the menstrual cycle. Hormonal contraceptive users and pregnant women completed the questionnaire, but their data were excluded from some of the menstrual cycle questions (see *Menstrual Function* in *Methods)*. The questionnaire asked about demographics, job role, menstrual function, exercise behaviors, eating behaviors, and psychological well-being. All participants provided informed consent. This study was approved by the Ministry of Defense Research Ethics Committee (REF: 2042/MODREC/21) and conducted in accordance with the Declaration of Helsinki (2013).

### Questionnaire

A custom-designed questionnaire was completed that contained up to 171 questions and took approximately 30 min to complete (Supplementary material). The questionnaire was a mix of validated questionnaires and some custom questions. Most questions were multiple choice with a few free text answers. The questionnaire was divided into: 1) demographics; 2) job role; 3) risk of eating disorders; 4) exercise behaviors, illness, and injury; 5) menstrual function; 6) bone health; 7) anxiety; 8) depression; 9) stress; 10) resilience; and 11) physical performance. The questionnaire addressed the components of the Triad (1) and RED-S (4) frameworks—menstrual disturbances, bone health, disordered eating, injury and illness, mood, and physical performance. Injury, illness, bone health, and physical performance are beyond the scope of this paper and only menstrual function, demographic, job role, risk of eating disorders, exercise behaviors, and psychological well-being data are presented here.

### Demographics and job role

Participants provided service, age, height, body mass, ethnicity, smoking status, job role, rank, length of service, number of deployments, pregnancy status, and history of hormonal contraceptive use. Servicewomen can enter all job roles in the military, which we broadly categorized as combat (roles directly involved in combat and considered the most physically arduous, e.g., infantry), combat support (roles that provide direct operational support to combat, but are typically less arduous than combat roles, e.g., artillery), and combat service support (roles that provide indirect logistical support to those in combat and are typically the least arduous roles, e.g., medical roles). Servicewomen were also categorized as Regulars (in full-time military service) or Reserves (typically part-time).

### Menstrual function

Menstrual function was measured using questions from the Low Energy Availability in Females–Questionnaire (LEAF-Q) (15). Current menstrual status was classified as oligomenorrhoea *or* amenorrhoea (grouped together) if < 9 menses in the last 12 months (2, 16). The number of menstrual cycles in the last 12 months is a common method for classifying menstrual status (16–23) and our definition is recommended by the Triad Consensus Screening Panel (2). History of secondary amenorrhoea was defined as ever having three consecutive months without a menstrual cycle in the absence of pregnancy or hormonal contraceptive use (16, 17, 19). Delayed menarche was defined as age of menarche at 15 years or older (2). Participants were excluded from the *current* menstrual status data if they used a hormonal contraceptive in the past 12 months, were pregnant, or failed to provide hormonal contraceptive or pregnancy status; hormonal contraceptive users and those who were pregnant were included in the *history of secondary amenorrhoea* and *delayed menarche* data.

### Exercise behaviors

Exercise behaviors were assessed by questions from the LEAF-Q (15) and custom questions. Exercise behavior questions were expanded to include exercise behaviors in the military and for recreation. For military exercise behaviors, participants provided the number of days in the last 12 months spent on field exercise, the number of days in the last 12 months spent on an arduous military training course, and the number of hours per week spent conducting military physical training. Field exercises are military training exercises that simulate combat or other military scenarios and typically involve load carriage, sleep loss, psychological stress, and restricted food intake. Similarly, arduous military courses are physically and mentally arduous, typically include some field exercises, and involves constant assessment either for a qualification or promotion to more senior ranks. Military physical training involves traditional aerobic and resistance training, and military specific training (e.g., load carriage). Participants also provided the number of hours per week spent personal physical training (e.g., running, cycling, resistance training), level of sport (for unit, regiment, or service), and number of hours per week of sport. The volume of each type of activity was quantified rather than providing a total training load due to the diversity of activities.

### Risk of eating disorders

Risk of eating disorders were assessed by the Female Athlete Screening Tool (FAST) (24) and Brief Eating Disorder in Athletes Questionnaire (BEDA-Q) (25). Some questions from the FAST were reworded to refer to military performance and training rather than athletic training and competitions. A score of 79 to 94 indicates subclinical disordered eating and > 94 indicates a clinical eating disorder (24). Risk of eating disorders was also measured using the BEDA-Q with a weighted BEDA-Q score of > 0.27 indicating high risk of an eating disorder (25).

### Psychological well-being

Psychological well-being was assessed using the 7-item Generalized Anxiety Disorder Assessment (GAD-7) (26), the 9-item Patient Health Questionnaire (PHQ-9) (27), the Perceived Stress Scale (28), and 10-item Connor-Davidson Resilience Scale (CD-RISC-10) (29). Anxiety and depression were scored according to severity using the respective GAD-7 (minimal, mild, moderate, severe) and PHQ-9 scores (minimal, mild, moderate, moderately severe, severe). A participant was defined as having anxiety if they scored ≥ 10 on the GAD-7 (26) and depression if they scored ≥ 10 on the PHQ-9 (27).

### Statistical analyses

Sample size was calculated based on determining the prevalence of oligomenorrhoea or amenorrhoea using the formula: ${{\text{Z}}_{1-\text{α}/2}}^{2}\text{p(1-p)}/{\text{d}}^{2}$, where Z_1−α/2_ is 1.96 for 5% type 1 error, p is the estimated population prevalence, and d is the marginal error. Assuming a prevalence of 11% for menstrual disturbances (16), it was estimated that 602 participants would be required for a marginal error of 2.5%. It was anticipated that 75% of Servicewomen will be aged 45 years or younger, 57% will be taking hormonal contraceptives, and 24% of Servicewomen will respond (unpublished internal data). Therefore, the survey was distributed to all Servicewomen under 45 years to allow the sample size to be met (~18,000).

All data were cleaned and analyzed in the R programming language (*v*. 4.1.2). The prevalence of demographic outcomes, menstrual disturbances (current oligomenorrhoea or amenorrhoea and history of amenorrhoea), exercise behaviors outcomes, risk of eating disorders (BEDA-Q and FAST risk), anxiety (GAD-7 score), depression (PHQ-9 score), stress (perceived stress score), and resilience (CD-RISC 10 score) were analyzed descriptively. Inferential statistics were performed with binary logistic regressions (glm function) to assess associations with two outcomes: current oligomenorrhoea or amenorrhoea (Yes = 1, No = 0) and history of amenorrhoea (Yes = 1, No = 0). The first model used demographic and job role data as predictor variables: age, height, body mass, service, job role, rank, length of service, and number of deployments. Demographic data were entered in the first model to identify the groups of women most at risk without cofounding for expected risk factors. The second model used exercise and sleep behaviors as predictor variables controlling for age and body mass: days on field exercise, days on arduous military training courses, level of sport, volume of military physical training, volume of personal physical training, hours of sleep, age, and body mass. All exercise data were entered in a second separate model to examine whether military or personal exercise behaviors were independent risk factors. The remaining six models used risk of eating disorders or psychological stress disorders as predictor variables—with separate models run for BEDA-Q risk, FAST risk, anxiety, depression, stress, and resilience—controlling for age, body mass, volume of military physical training, and volume of personal physical training. Measures of eating disorders and psychological stress disorders were analyzed in separate models due to likely collinearity, which makes coefficients difficult to interpret. Anxiety and depression were entered as binary diagnoses based on their scorings. The measures of eating disorders and psychological stress disorders were entered in the remaining models to examine the independent effects of these known risk factors controlling for demographic and exercise behaviors. Most predictor variables were categorical with the first or lowest category used as the reference category; deployments, perceived stress score, and CD-RISC-10 score were integers. Eight logistic regression models were run for each of the two outcomes and so the alpha was corrected to *p* ≤ 0.006 (0.05/8).

## Results

### Participants

**Figure 1:**
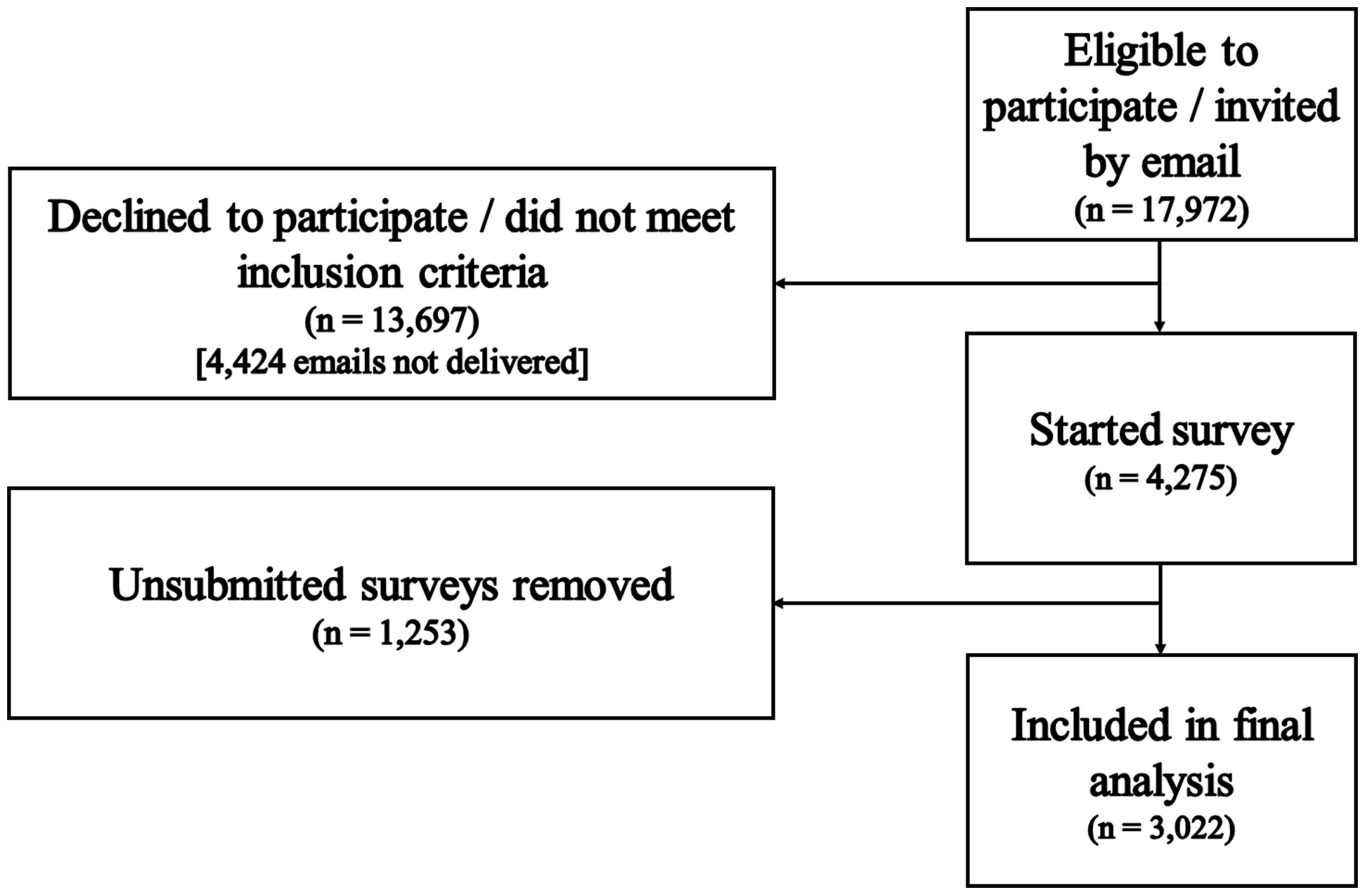
Survey sampling.

Participant flow through the study is shown in Figure 1. A total of 17,972 women were eligible to participate with 3,022 completing the survey (16.8% response rate). A total of 2,665 participants completed the survey in response to the email and 357 in response to other adverts. Demographic data for the final sample are shown in Table 1.

**Table 1:** Participant demographics (*n* = 3,022).

**Measure**	***n* (%) or median (interquartile range)**
**Age (years)**	
17–24	468 (16%)
25–29	647 (21%)
30–34	683 (23%)
35–39	662 (22%)
40–44	555 (18%)
**Height (m)**	
< 1.60	555 (18%)
1.60–1.69	1,544 (51%)
1.70–1.79	819 (27%)
≥ 1.80	98 (3%)
**Body mass (kg)**	
< 60	519 (17%)
60–69	1,133 (38%)
70–79	803 (27%)
80–89	365 (12%)
≥ 90	193 (6%)
**Ethnicity**	
White	2,824 (94%)
Black/African/Caribbean	81 (3%)
Asian	20 (1%)
Mixed/Multiple ethnicities	63 (2%)
Other	8 (< 1%)
**Smokers**	228 (8%)
**Hormonal contraceptive users**	1,424 (47%)
**Pregnant**	138 (5%)
**Service**	
Army	1,341 (45%)
Royal navy	554 (18%)
Royal air force	1,118 (37%)
**Rank**	
Junior non-commissioned officer and below	1,235 (41%)
Senior non-commissioned officer	729 (24%)
Junior officer	597 (20%)
Senior officer	446 (15%)
**Job role**	
Combat	141 (5%)
Combat support	1,024 (34%)
Combat service support	1,855 (61%)
**Regular or reserve service**	
Regular	2,802 (93%)
Reserve	201 (7%)
**Length of service (years)**	
≤ 5	937 (31%)
6–10	648 (21%)
11–15	597 (20%)
16–20	(6%)
> 20	370 (12%)
**Career deployments**	2 (1, 5)
**Typical sleep duration (hours)**	
≤ 5	353 (12%)
6–7	1,983 (66%)
≥ 8	680 (23%)

Missing data: Service, n = 9; Age (years), n = 7; Height (m), n = 6; Body Mass (kg), n = 9; Ethnicity, n = 26; Smokers, n = 3; Current Hormonal Contraceptive User, n = 11; Currently Pregnant, n = 33; Rank, n = 15; Job Role, n = 2; Regular or Reserve Service, n = 19; Length of Service (Years), n = 1; Number of Career Deployments, n = 20; Typical Sleep Duration (hours), n = 6.

Data are n (%) or median (interquartile range).

### Menstrual function

Menstrual function data can be seen in Table 2. A total of 18% of women had a current menstrual disturbance (oligomenorrhoea or amenorrhoea), 25% of women had a history of amenorrhoea, and 14% of women had delayed menarche.

**Table 2:** Menstrual function (*n* = 3,022).

**Measure**	***n* (%) or median (interquartile range)**
**Menstrual status^a^**	
Eumenorrheic	1,181 (82%)
Oligomenorrheic/amenorrhoeic	260 (18%)
**History of amenorrhoea**	738 (25%)
**Delayed menarche**	
Unsure	99 (3%)
No	2,494 (83%)
Yes	425 (14%)

a n = 1,441; 1,581 women were excluded because they used a hormonal contraceptive in the past year and/or were pregnant and/or did not provide hormonal contraceptive or pregnancy status and/or did not answer the related question.

Missing data: Menstrual Status, n = 1,581; History of Oligomennorrhoea / Amenorrhoea, n = 18; Delayed Menarche, n = 4.

Data are n (%).

### Exercise behaviors, risk of eating disorders, and psychological well-being

Exercise behaviors, risk of eating disorders, and psychological well-being data are presented in Table 3. Most women had not been on a field exercise (65%) or completed an arduous military training courses (83%) in the last 12 months and did not play sport (63%). Most women conducted < 1 h·week^−1^ of military physical training (43%) and 1 to 3 h·week^−1^ of personal physical training (38%), and slept 6 to 7 h per night (67%). A total of 13% of participants were high risk of an eating disorder according to the BEDA-Q and FAST scores, with a further 34% at risk of disordered eating according to the FAST score. A total of 25% of participants had symptoms of anxiety (GAD-7 score ≥ 10) and 26% had symptoms of depression (PHQ-9 score ≥ 10).

**Table 3:** Exercise behaviors, risk of eating disorders, and psychological well-being (*n* = 3,022).

**Measure**	***n* (%) or Median (interquartile range)**
**Field exercise (days in last 12 months)**	
0	1,960 (65%)
1–10	556 (8%)
> 10	494 (16%)
**Arduous military courses (days in last 12 months)**	
0	2,504 (83%)
1–10	366 (2%)
> 10	129 (4%)
**Military physical training (h·week** ^**−1**^ **)**	
< 1	1,298 (43%)
1–3	986 (33%)
3–5	569 (19%)
> 5	145 (5%)
**Personal physical training (h·week** ^**−1**^ **)**	
< 1	385 (13%)
1–3	1,149 (38%)
3–5	834 (28%)
> 5	639 (21%)
**Military sport (level)**	
None	1,887 (63%)
Unit/corps/region	565 (19%)
Service	562 (19%)
**Military sport (h·week** ^**−1**^ **)**	
< 1	176 (16%)
1–3	317 (28%)
3–5	296 (6%)
> 5	333 (30%)
**BEDA-Q risk**	
Low	2,581 (87%)
High	383 (13%)
**FAST score**	77 (67, 88)
**FAST risk**	
None	1,449 (52%)
Subclinical disordered eating	957 (34%)
Eating disorder	373 (13%)
**GAD-7 score**	5 (2, 9)
**Anxiety**	
Minimal	1,293 (43%)
Mild	948 (32%)
Moderate	441 (15%)
Severe	293 (10%)
**PHQ-9 score**	5 (2, 10)
**Depression**	
Minimal	1,362 (46%)
Mild	845 (28%)
Moderate	428 (14%)
Moderately severe	221 (7%)
Severe	122 (4%)
**PSS score**	18 (12, 22)
**CD-RISC-10 score**	27 (22, 31)

BEDA-Q, Brief Eating Disorder in Athletes Questionnaire; CD-RISC-10, Connor-Davidson Resilience Scale; FAST, Female Athlete Screening Tool; GAD-7, Generalized Anxiety Disorder Assessment; PHQ-9, Patient Health Questionnaire; PSS, Perceived Stress Scale.

Missing data: Days of Field Exercise in Last 12 Months, n = 12; Days on Arduous Military Training Course in Last 12 Months, n = 23; Volume of Military Physical Training (hours per week), n = 24; Volume of Personal Physical Training (hours per week), n = 15; Military Sport (Level), n = 8; Volume of Sport (hours per week), n = 1,900; BEDA-Q risk, n = 58; FAST Score, n = 243; FAST Risk, n = 243; GAD Score, n = 47; Anxiety, n = 47; PHQ-9 Score, n = 44; Depression, n = 44; PSS Score, n = 50, CD-RISC-10 Score, n = 27.

Data are n (%) or median (interquartile range).

### Risk factors for menstrual dysfunction

Associations between demographics and menstrual disturbances are shown in Table 4. Women aged 35 to 39 years and 40 to 44 years were at lower risk of oligomenorrhoea or amenorrhoea compared with women aged 17 to 24 years (*p* ≤ 0.003). Women aged 40 to 44 years were at a lower risk of a history of amenorrhoea compared with women aged 17 to 24 years (*p* = 0.004). Women who sleep ≥ 8 h were at a lower risk of a history of amenorrhoea compared with women who sleep ≤ 5 h (*p* = 0.006).

**Table 4:** Associations between demographics and menstrual function.

	**Oligomenorrhoea/amenorrhoea**			**History of amenorrhoea**		
**Measure^a^**	**Prevalence (%)**	**OR (95% CI)**	**p**	**Prevalence (%)**	**OR (95% CI)**	**p**
**Age (years)**						
17–24	32	—	—	32	—	—
25–29	22	0.56 (0.35, 0.90)	0.018	27	0.89 (0.67, 1.19)	0.436
30–34	20	0.52 (0.30, 0.90)	0.019	26	0.90 (0.64, 1.26)	0.534
35–39	11	0.22 (0.11, 0.43)	**<0.001**	22	0.71 (0.47, 1.07)	0.102
40–44	13	0.32 (0.15, 0.68)	**0.003**	17	0.49 (0.30, 0.79)	**0.004**
**Body mass (kg)**						
< 60	20	—	—	29	—	—
60–69	20	1.11 (0.75, 1.66)	0.615	26	0.91 (0.71, 1.15)	0.418
70–79	15	0.83 (0.53, 1.30)	0.404	22	0.76 (0.58, 0.98)	0.035
80–89	16	1.04 (0.61, 1.76)	0.886	20	0.68 (0.49, 0.95)	0.024
More than 90	18	1.12 (0.60, 2.05)	0.715	23	0.83 (0.55, 1.23)	0.361
**Service**						
Army	17	—	—	26	—	—
Royal navy	17	0.88 (0.59, 1.31)	0.542	22	0.80 (0.62, 1.02)	0.076
Royal air force	20	1.16 (0.83, 1.60)	0.380	24	0.91 (0.75, 1.11)	0.366
**Rank**						
Junior NCO and below	22	—	—	29	—	—
Senior NCO	15	0.99 (0.63, 1.58)	0.981	21	0.75 (0.56, 1.00)	0.054
Junior officer	20	1.01 (0.69, 1.48)	0.949	26	0.93 (0.73, 1.18)	0.547
Senior officer	11	0.74 (0.41, 1.30)	0.302	18	0.75 (0.54, 1.05)	0.096
**Job role**						
Combat	12	—	—	21	—	—
Combat support	19	1.49 (0.69, 3.60)	0.341	23	0.99 (0.63, 1.59)	0.957
Combat service support	18	1.52 (0.72, 3.62)	0.303	26	1.26 (0.81, 1.99)	0.319
**Length of service (years)**						
5 or less	25	—	—	30	—	—
6–10	20	1.01 (0.64, 1.60)	0.960	25	0.85 (0.64, 1.12)	0.245
11–15	16	1.09 (0.61, 1.95)	0.774	22	0.82 (0.57, 1.17)	0.270
16–20	12	1.05 (0.51, 2.16)	0.905	21	1.02 (0.65, 1.61)	0.930
More than 20	12	0.93 (0.41, 2.12)	0.862	18	0.94 (0.55, 1.59)	0.819
**Career deployments**	—	0.026^b^	0.124	—	0.022^b^	0.073
**Typical sleep (hours per night)**						
5 or less	20	—	—	29	—	—
6–7	18	0.83 (0.54, 1.29)	0.398	25	0.79 (0.61, 1.04)	0.087
8 or more	18	0.74 (0.45, 1.22)	0.232	22	0.65 (0.48, 0.89)	**0.006**

CI, confidence interval; NCO, non-commissioned officer; OR, odds ratio.

a Controlling for all other factors in the table.

b Coefficient not odds ratio.

Associations between exercise behaviors and menstrual disturbances are shown in Table 5. Exercise behaviors were not associated with current oligomenorrhoea or amenorrhoea. Women completing > 10 days of field exercise in the last 12 months were at higher risk of history of amenorrhoea than women completing 0 days of field exercise (*p* = 0.004).

**Table 5:** Associations between exercise behaviors and menstrual function.

	**Oligomenorrhoea/amenorrhoea**			**History of amenorrhoea**		
**Measure^a^**	**Prevalence (%)**	**OR (95% CI)**	**p**	**Prevalence (%)**	**OR (95% CI)**	**p**
**Field exercise (days in last 12 months)**						
0	17	—	—	22	—	—
1–10	19	0.88 (0.58, 1.31)	0.541	26	1.12 (0.88, 1.42)	0.350
More than 10	21	1.08 (0.71, 1.63)	0.710	32	1.45 (1.13, 1.85)	**0.004**
**Arduous military courses (days in last 12 months)**						
0	17	—	—	24	—	—
1–10	20	0.97 (0.63, 1.49)	0.907	26	0.91 (0.69, 1.20)	0.512
More than 10	24	1.06 (0.51, 2.10)	0.867	34	1.17 (0.78, 1.75)	0.445
**Military physical training (h·week** ^**−1**^ **)**						
< 1	18	—	—	23	—	—
1–3	17	0.97 (0.69, 1.35)	0.837	24	1.09 (0.89, 1.34)	0.409
3–5	19	1.03 (0.69, 1.53)	0.887	28	1.24 (0.97, 1.58)	0.080
> 5	23	1.06 (0.54, 1.97)	0.867	27	1.00 (0.66, 1.49)	0.993
**Personal physical training (h·week** ^**−1**^ **)**						
< 1	18	—	—	24	—	—
1–3	13	0.64 (0.41, 1.04)	0.064	21	0.80 (0.60, 1.06)	0.120
3–5	19	0.94 (0.58, 1.53)	0.794	24	0.95 (0.71, 1.28)	0.725
> 5	26	1.31 (0.80, 2.17)	0.290	31	1.24 (0.91, 1.69)	0.173
**Military sport (level)**						
None	17	—	—	23	—	—
Unit/corps/region	18	0.94 (0.63, 1.37)	0.746	26	1.03 (0.82, 1.29)	0.798
Service	21	1.11 (0.77, 1.58)	0.576	27	1.13 (0.90, 1.42)	0.284

CI, confidence interval; OR, odds ratio.

a Controlling for all other factors in the table as well as age and body mass.

Associations between risk of eating disorders and psychological well-being with menstrual disturbances are shown in Table 6. Women at high risk of an eating disorder (based on FAST score) were at higher risk of oligomenorrhoea or amenorrhoea (*p* = 0.002). Women at high risk of an eating disorder (based on BEDA-Q score) or high risk of subclinical disordered eating or an eating disorder (based on FAST score) and women with anxiety or depression were at a higher risk of a history of amenorrhoea (*p* < 0.001).

**Table 6:** Associations between risk of eating disorders and psychological well-being with menstrual function.

	**Oligomenorrhoea/amenorrhoea**			**History of amenorrhoea**		
**Measure^a^**	**Prevalence (%)**	**OR (95% CI)**	**p**	**Prevalence (%)**	**OR (95% CI)**	**p**
**BEDA–Q risk**						
Low	17	—	—	23	—	—
High	26	1.66 (1.09, 2.47)	0.015	32	1.52 (1.19, 1.94)	**<0.001**
**FAST risk**						
None	14	—	—	20	—	—
Subclinical disordered eating	21	1.56 (1.13, 2.17)	0.008	27	1.50 (1.23, 1.83)	**<0.001**
Eating disorder	29	1.97 (1.26, 3.04)	**0.002**	36	2.14 (1.63, 2.79)	**<0.001**
**Anxiety**						
No	17	—	—	22	—	—
Yes	22	1.20 (0.87, 1.64)	0.258	31	1.46 (1.20, 1.77)	**<0.001**
**Depression**						
No	16	—	—	22	—	—
Yes	23	1.29 (0.94, 1.75)	0.113	31	1.48 (1.22, 1.79)	**<0.001**
**PSS score^b^**	—	0.02 (0.00, 0.04)	0.050	—	0.02 (0.00, 0.03)	0.009
**CD–RISC−10 score^b^**	—	−0.01 (−0.03, 0.01)	0.356	—	−0.01 (−0.02, 0.01)	0.386

CD–RISC−10, Connor–Davidson Resilience Scale; CI, confidence interval; OR, odds ratio; PSS, Perceived Stress Scale.

a Each measure was run in separate models controlling for age, body mass, volume of military physical training, and volume of personal physical training.

b Coefficient and 95% confidence intervals not odds ratio.

## Discussion

This study investigated the prevalence of menstrual disturbances and associated risk factors in a large cohort of British Servicewomen. Servicewomen are exposed to multiple risk factors for menstrual disturbances and the Triad/RED-S including high exercise volumes, restricted energy intake, psychological stress, and poor sleep (5, 6), but the prevalence of menstrual disturbances and associated risk factors were yet to be studied in the UK Armed Forces. Menstrual disturbances are positively associated with the risk of bone stress injuries, musculoskeletal injuries, and low bone mass [reviewed in O'Leary (6)] and so these data have important clinical policy implications for the military. The large sample size and homogenous population also make these data useful for managing health in young active women.

### Prevalence of menstrual disturbances

Eighteen percent of women had oligomenorrhoea or amenorrhoea, 25% of women had a history of amenorrhoea, and 14% of women had delayed menarche. The prevalence of secondary amenorrhoea is 2 to 5% in non-athletes of reproductive age (3, 14, 30, 31), but oligomenorrhoea was not reported in these studies making comparisons with our data difficult. Studies that have investigated oligomenorrhoea or amenorrhoea in non-athletes report a prevalence of 11 to 14% (31, 32) suggesting a slightly higher prevalence of oligomenorrhoea and amenorrhoea in Servicewomen than the general population. Similarly, we found a higher prevalence of delayed menarche than is reported in the UK general population (10%) (33), likely due to the self-selection of active women to military service. The prevalence of menstrual disturbances is higher in athletes than the general population, but varies widely by athlete group (14). Endurance athletes (recreational and elite) had a prevalence of 21 to 43% of oligomenorrhoea or amenorrhoea using similar criteria to this study (17, 19–23), but a higher prevalence has been reported (above 60%) in aesthetic or “lean” sports and activities like dance and long-distance running (3, 14). These athlete data are typically in young or adolescent women whereas our data included women up until age 44 years, and older age was associated with a lower prevalence of menstrual disturbances in agreement with athlete data (14). The prevalence of oligomenorrhoea or amenorrhoea in the youngest women in our study (17 to 24 years) was 32%, similar to the young athlete groups presented here. Comparisons between studies should be made with caution due to inconsistencies with defining and measuring menstrual function, and studies in athletes are typically small, subject to recruitment bias, and at risk of overestimating menstrual disturbances. Nevertheless, our data show that the youngest group of Servicewomen in our study experience a similar prevalence of menstrual disturbances and delayed menarche to young female athletes.

Several studies have investigated menstrual disturbances in military populations (9, 16, 34–38), but our data are the first across the entire UK Armed Forces. Cross-sectional studies from the 1990s in the US Army identified the prevalence of oligomenorrhoea or amenorrhoea (< 9 cycles in 12 months or 3 months with no cycle) as 12% in the last 24 months with delayed menarche in 13% (≥ 15 years) (16) and the prevalence of ever having amenorrhoea (> 6 months without menses) as 15% with delayed menarche in 4% (≥ 17 years) (35). It is unclear why we report a higher prevalence of menstrual disturbances than these studies in the US Army, but differences in population, methods, and definitions of menstrual function likely contribute. The prevalence of menstrual disturbances is higher in basic military training than in trained Servicewomen, with more than 65% of women undergoing basic military training reporting menstrual cycle disturbances or changes (9, 34, 36, 38). Basic military training is one the most arduous part of a military career (12) and likely explains the higher prevalence of menstrual disturbances in these studies.

### Risk factors for menstrual disturbances

Older age was associated with a lower prevalence of oligomenorrhoea or amenorrhoea. Younger women (within ~5 years of menarche) and older women (above a gynecological age of ~30 years) are at increased risk of menstrual disturbances (14). Almost all the women in this study had a gynecological age < 30 years and so the higher prevalence of oligomenorrhoea or amenorrhoea in younger women could be due to being closer to age of menarche. The HPO axis may also become more resilient to stressors with age (39). Older women are also more likely to be in senior roles and complete fewer physically demanding military activities. Older women were also at a lower risk of ever having amenorrhoea despite having had more time to develop menstrual disturbances, although length of service was controlled for in our analyses. Women aged over 40 years could be a healthy survivor cohort; women suffering from menstrual disturbances may be more likely to leave the military because of the stresses causing those menstrual disturbances or because a menstrual disturbance increases the risk of musculoskeletal injury or other health outcome. The other demographic factor associated with a menstrual disturbance (history of amenorrhoea) was shorter sleep duration. Sleep was not associated with a current menstrual disturbance, but habitual short sleep can activate the HPA axis (5, 9) and increase risk of ever having amenorrhoea. We are unable to confirm the direction of this relationship and poor sleep might be an indicator of psychological ill health. Sleep duration (and other risk factors) may be associated with a history of amenorrhoea but not a current menstrual disturbance due to a greater number of participants included in the history of amenorrhoea analysis increasing statistical power; all participants were included in the analysis of history of amenorrhoea whereas only those not using hormonal contraceptives were included in the current menstrual disturbance analyses.

Exercise behaviors were not associated with current oligomenorrhoea or amenorrhoea, but a higher total volume of field exercise was associated with increased risk of ever having amenorrhoea. Most Servicewomen did not complete any field exercise (typical of some less arduous jobs) whereas 8% completed 1 to 10 days (typically one field exercise) and 16% completed more than 10 days (likely several field exercises). Completing more than 10 days of field exercise is likely indicative of a more physically and psychologically demanding job; however, job role and other exercise measures were not associated with a menstrual disturbance. Field exercises are categorized by high energy expenditures, restricted food intake, sleep restriction, and psychological stress, which could all contribute to suppression of the HPO axis (6, 8–10), although volume of field exercise was not associated with a current menstrual disturbance. Total exercise volume in the military results from a combination of occupational requirements (i.e., field exercises, training courses, physical training) and personal exercise. Here we provide evidence that occupational [field] exercise volume was associated with menstrual history, but other exercise behaviors were not.

Risk of an eating disorder was associated with oligomenorrhoea or amenorrhoea (based on FAST score) and history of amenorrhoea (based on BEDA-Q and FAST score). A total of 13% of participants were at high risk of an eating disorder measured by both BEDA-Q and FAST, with a further 34% at risk of disordered eating measured by the FAST. Eating behaviors were scored by the BEDA-Q and FAST to capture eating disorders specific to active women, with our data suggesting the FAST may be more sensitive for detecting menstrual disturbances. The prevalence of behaviors indicative of eating disorders reported by female athletes varies widely (6 to 45%), but is higher in women than men, athletes than non-athletes, and lean than non-lean sports (40, 41). Data from active duty US Servicewomen shows 34% are at risk of disordered eating (16). Comparisons between studies is difficult due to differences in the screening tool used. Eating disorders and/or disordered eating are recognized as a common cause of menstrual disturbances in the Triad (1, 2) and RED-S (3, 4) frameworks, and here we provide evidence of a similar association in British Servicewomen. The mechanism may be low energy availability, but eating disorders increase the risk of psychological ill health (18) (or are caused by psychological ill health) and are stress disorders that activate the HPA axis (42). Unlike some sports, a specific body composition is not a prerequisite to successful military performance, although some militaries have body composition standards. Anxiety and depression were associated with a history of amenorrhoea, and mood disorders contribute to menstrual disturbances (42). Similar findings from the US Air Force show that life, but not work, psychological stress was associated with irregular cycle lengths in civilian and military women (37). The direction of this relationship is not clear as low energy availability increases risk of mood disturbances and mood disturbances may increase the risk of eating disorders and low energy availability (4).

### Limitations

This study is subject to overestimation of menstrual disturbances due to self-selection of participants. We attempted to reduce the risk of this bias by offering renumeration and by advertising the study across multiple platforms. Our data are likely biased by the fact that deployed women were less likely to participate in this study. A large proportion of our participants took hormonal contraceptives, and it is unclear how excluding hormonal contraceptive users biases our data. We are unable to confirm functional hypothalamic amenorrhoea with our methods and subtle menstrual disturbances would have been missed. We did not have a group of non-military participants and so it is not clear whether a military career increases the risk of menstrual disturbances. Finally, our measures are all self-reported and subject to recall error or bias.

### Conclusions

British Servicewomen had a similar prevalence of menstrual disturbances to groups of endurance athletes. Younger age, shorter habitual sleep duration, higher volumes of field exercise, risk of eating disorders, and psychological ill health were associated with increased risk of menstrual disturbances. These risk factors provide unique targets for the protection of the reproductive axis in Servicewomen.

## Data availability statement

The datasets presented in this article are not readily available because approval must be sought from the UK Ministry of Defense. Requests to access the datasets should be directed to thomas.oleary100@mod.gov.uk.

## Ethics statement

The studies involving human participants were reviewed and approved by Ministry of Defense Research Ethics Committee (MODREC). All participants provided informed consent.

## Author contributions

TO'L: conceptualization, data curation, formal analysis, software, writing—original draft, and funding acquisition. CP, NK, and SW: conceptualization and writing—reviewing and editing. CC: conceptualization, data curation, and writing—reviewing and editing. RD: data curation and writing—reviewing and editing. JG: conceptualization, writing—reviewing and editing, and funding acquisition. All authors contributed to the article and approved the submitted version.

## Funding

This study was funded by the British Army, UK Ministry of Defense.

## Conflict of interest

The authors declare that the research was conducted in the absence of any commercial or financial relationships that could be construed as a potential conflict of interest.

## Publisher's note

All claims expressed in this article are solely those of the authors and do not necessarily represent those of their affiliated organizations, or those of the publisher, the editors and the reviewers. Any product that may be evaluated in this article, or claim that may be made by its manufacturer, is not guaranteed or endorsed by the publisher.
