# Supplementary material for: Menstrual disturbances in British Servicewomen: A cross-sectional observational study of prevalence and risk factors
Source: Front Nutr. 2022 Oct 19;9:984541. doi: 10.3389/fnut.2022.984541 (PMC9626978; doi:10.3389/fnut.2022.984541)
Supplement: Supplementary file 1 [file Data_Sheet_1.PDF]

Section A: About You

This section will ask you questions about yourself, such as your height, ethnicity and smoking habits. For ‘free text answer options please do not include any identifying personal information.

1. **What is your age?**

Scale (years): 16 – 19; 20 – 24; 25 – 29; 30 – 34; 37 – 37; 38 – 40; 41 - 44

2. **What is your height?**

Scale (cm): Less than 150; 150 – 154; 155 – 159; 160 – 164; 165 – 169; 170 – 174; 175 – 179; 180 – 184 – 185 – 189; 190 or above.

3. **What is your weight?**

Scale (kg): Less than 45; 45 – 49; 50 – 54; 55 – 59; 60 – 64; 65 – 69; 70 – 74; 75 – 79; 80 – 84; 85 – 89; 90 – 94; 95 – 99; 100 or greater

4. **What was your lowest adult weight?** (an adult is classified as anyone who is aged 18 years old and over)

Scale (kg): Less than 45; 45 – 49; 50 – 54; 55 – 59; 60 – 64; 65 – 69; 70 – 74; 75 – 79; 80 – 84; 85 – 89; 90 – 94; 95 – 99; 100 or greater

5. **How old were you when you were at your lowest adult weight?**

Scale (years): 16 – 19; 20 – 24; 25 – 29; 30 – 34; 37 – 37; 38 – 40; 41 - 44

6. **What was your highest adult weight?** (please do not include your weight during pregnancy)

Scale (kg): Less than 45; 45 – 49; 50 – 54; 55 – 59; 60 – 64; 65 – 69; 70 – 74; 75 – 79; 80 – 84; 85 – 89; 90 – 94; 95 – 99; 100 or greater

7. **What is your ethnic group?**

Scale: White; Asian; Black/African/Caribbean; Mixed/Multiple ethnicities; Other; Unknown/prefer not to say

8. **Are you pregnant or currently nursing?**

Scale: Yes/No

9. **If yes to 8, how many previous full-term pregnancies have you had?**

Scale: 1; 2; 3; 4; 5 or more

10. **Do you currently smoke?** (Smoking is defined as someone who smokes a tobacco product at least once a day)?

Scale: Yes/No

11. **If yes to 10, how many per day?**

Scale: 1 - 5; 5 - 10; 11 – 15; 16 or more

12. **(If no to 10) Did you previously smoke?**

Scale: Yes/No

13. **(If yes to 12) When did you stop?**

Scale: Within the last 3 months; Within the last 12 months; Over 1 year ago; Over 2 years ago

Section B: About Your Job

For ‘free text answer options please do not include any identifying personal information.

14. How long have you been in the military?

Scale: Less than a year; 1- 5 years; 6 – 10 years; 11 – 15 years; 16 – 20 years; 21- 25 years

15. What is your service?

Scale: Royal Navy; Royal Marines; Army; Royal Air Force

16. Are you in Regular or Reserve Service?

Scale: Regular; Reserve; Full Time Reserve Service

17. Have you had a break in Service?

Scale: Yes; No

18. What is your rank?

Scale: (Army): Recruit; Private; Lance Corporal; Corporal; Sergeant; Staff Sergeant; Warrant Officer 1; Warrant Officer 2; Officer Cadet; Second Lieutenant; Lieutenant; Captain; Major; Lieutenant Colonel and above

Scale: (Royal Navy): Recruit; Able Rate; Seaman; Leading Hand; Petty Officer; Chief Petty Officer; Warrant Officer 1; Warrant Officer 2; Officer Cadet; Midshipman; Sub Lieutenant; Lieutenant; Lieutenant Commander; Commander and above

Scale: (Royal Air Force): Recruit; Aircraftman; Senior Aircraftman; Corporal; Sergeant; Chief Technician; Flight Sergeant; Warrant Officer; Officer Cadet; Pilot Officer; Flying Officer; Flight Lieutenant; Squadron Leader; Wing Commander and above

Scale: (Royal Marines): Recruit; Marine; Lance Corporal; Corporal; Sergeant; Colour Sergeant; Warrant Officer 1; Warrant Officer 2; Second Lieutenant; Lieutenant; Captain; Major; Lieutenant Colonel and above

19. What is your career employment group?

Scale: Engineering; Logistics; Combat/Warfare; Intelligence, IT, and Communications; HR, Finance, Personnel Support, and Education Training Services; Medical; Music and Ceremonial/ Royal Marines Band; Aviation and Air Crew; Air Operations Support; Chaplaincy; Force Protection; Other

20. Are you medically fit to deploy (MFD)?

Scale: Yes/No

21. (If No to 20) Please specify why?

Scale: Musculoskeletal injury; Medical condition; Prefer not to say; Other

22. How physically hard do you find your job?

Scale: Very very light; very light; fairly light; somewhat hard; hard; very hard; very very hard.

23. How many hours do you typically sleep per night?

Scale: Less than 4; 4 – 5; 5 – 6; 6 – 7; 7 – 8; More than 8

24. How many days have you been on field exercises in the last 12 months?

Scale: 0; 1-10; 10-20; 21 and above

25. How many days have you been on physically arduous training courses in the last 12 months (e.g., Section or Platoon Commander's Battle Course)?

Scale: 0; 1-10; 10-20; 21 or more

26. How many deployments (overseas or domestic) have you been on in your career?

Scale: 0; 1; 2; 3; 4; 5; 6; 7; 8; 9; 10; Other (Please specify)

Section C: Eating Behaviours (FAST [30 to 62] / BDA-Q [64 to 72])
This section explores areas relating to your eating and exercise habits. It includes questions about diet, weight, allergies and exercise/training. For ‘free text answer options please do not include any identifying personal information.

27. Do you follow a vegetarian or vegan diet?
Scale: Yes/No

28. Do you have any food allergies, intolerances or restrict any food groups?
Scale: Yes/No

29. (If yes to 28), please specify?
Scale: Celery; Cereals containing gluten (barley, wheat, rye); Crustaceans (eg: crab, lobster, prawns); Egg; Fish; Milk/dairy/Lactose; Mustard; Nuts; Peanuts; Sesame seed; Soya; Sulphites; Other

30. I participate in additional recreational physical activity over 20 minutes in length on days that I have physical training or field exercise.
Scale: Frequently; Sometimes; Rarely; Never

31. If I cannot exercise, I find myself worrying that I will gain weight.
Scale: Frequently; Sometimes; Rarely; Never

32. I believe that most female soldiers have some form of disordered eating habit.
Scale: Strongly agree; Agree; Disagree; Strongly disagree

33. During training, I control my fat, carbohydrate, and calorie intake carefully.
Scale: Frequently; Sometimes; Rarely; Never

34. I will eat foods that have more than 3 grams of fat. (1 teaspoon of butter is 3g of fat)
Scale: Strongly agree; Agree; Disagree; Strongly disagree

35. I feel that my performance would improve if I lost weight.
Scale: Strongly agree; Agree; Disagree; Strongly disagree

36. If I weighed myself tomorrow and gained 2 pounds, I would exercise harder or longer than usual.
Scale: Frequently; Sometimes; Rarely; Never

37. I weigh myself:
Scale: Daily; 2 or more times a week; Weekly; Monthly or less

38. If I choose to complete additional exercise on a day of physical training or field exercise, I exercise for:
Scale: 2 or more hours; 1 – 2 hours; 45 – 60 minutes; 30-44 minutes; less than 30 minutes; I choose to do not additional exercise

39. If I know that I will be consuming alcoholic beverages, I will skip meals on that day or the following day.
Scale: Frequently; Sometimes; Rarely; Never

40. I feel guilty if I choose fried foods for a meal.
Scale: Frequently; Sometimes; Rarely; Never

41. If I were injured, I would still exercise even if I were instructed not to do so by my physical training instructor or doctor.
Scale: Strongly agree; Agree; Disagree; Strongly disagree

42. I take dietary or herbal supplements to increase my metabolism and/or to assist in burning fat.
Scale: Frequently; Sometimes; Rarely; Never

43. I am concerned about my level of body fat.
Scale: Frequently; Sometimes; Rarely; Never

44. Being in the military, I am very conscious about consuming adequate calories and nutrients on a daily basis.
Scale: Frequently; Sometimes; Rarely; Never

45. I am worried that if I were to gain weight, my performance would decrease.
Scale: Strongly agree; Agree; Disagree; Strongly disagree

46. I think that being thin is associated with my physical performance.
Scale: Strongly agree; Agree; Disagree; Strongly disagree

47. I train intensely for my job or sport to make sure I will not gain weight.
Scale: Frequently; Sometimes; Rarely; Never

48. Has the amount of food you eat changed since the initial COVID-19 'Lockdown' on 23rd March 2020?
Scale: No change; I eat a lot more now; I eat a little more now; I eat a little less now; I eat a lot less now

49. I choose to exercise on days off or "rest days".
Scale: Frequently; Sometimes; Rarely; Never

50. My friends tell me that I am thin, but I feel fat.
Scale: Frequently; Sometimes; Rarely; Never

51. I feel uncomfortable eating around others.
Scale: Frequently; Sometimes; Rarely; Never

52. I limit the amount of carbohydrates I eat.
Scale: Frequently; Sometimes; Rarely; Never

53. I try to lose weight to please others.
Scale: Frequently; Sometimes; Rarely; Never

54. If I were unable to train in my job, I would not feel good about myself.
Scale: Strongly agree; Agree; Disagree; Strongly disagree

55. If I were injured and unable to exercise, I would restrict my caloric intake.
Scale: Strongly agree; Agree; Disagree; Strongly disagree

56. In the past 2 years I have been unable to undertake my job due to an injury.
Scale: 7 or more times; 4-6 times; 1-3 times; No significant injuries

57. During work I have trouble concentrating due to feelings of guilt about what I have eaten that day.
Scale: Frequently; Sometimes; Rarely; Never

58. I feel that I have a lot of good qualities.
Scale: Strongly agree; Agree; Disagree; Strongly disagree

59. At times I feel that I am no good at all.
Scale: Strongly agree; Agree; Disagree; Strongly disagree

60. I strive for perfection in all aspects of my life.
Scale: Strongly agree; Agree; Disagree; Strongly disagree

61. I avoid eating meat to stay thin.
Scale: Strongly agree; Agree; Disagree; Strongly disagree

62. I am happy with my present weight.
Scale: Yes; No

63. I have done things to keep my weight down that I believe are unhealthy.
Scale: Frequently; Sometimes; Rarely; Never

64. I always feel guilty after overeating
Scale: Always; usually; often, sometimes, rarely, never

65. I am preoccupied with the desire to be thinner
Scale: Always; usually; often, sometimes, rarely, never

66. I think that my stomach is too big
Scale: Always; usually; often, sometimes, rarely, never

67. I feel satisfied with the shape of my body
Scale: Always; usually; often, sometimes, rarely, never

68. My parents have expected excellence of me
Scale: Always; usually; often, sometimes, rarely, never

69. As a child, I tried very hard to avoid disappointing my parents and teachers
Scale: Always; usually; often, sometimes, rarely, never

70. Are you trying to lose weight now?
Scale: Yes/No

71. Have you tried to lose weight previously?
Scale: Yes/No

72. (If yes to 71) How many times have you tried to lose weight?
Scale: 1-2; 3-5; over 5 times

73. Have you ever been diagnosed with an eating disorder e.g., Anorexia, bulimia?
Scale: Yes/No

Section D: Exercise Behaviours, Illness, and Injury (LEAF-Q [84 to 93])

This section explores areas relating to other aspects of your exercise habits, injuries and illness. It includes questions about what type of exercise/training you do and how often, and exercise/training associated with your job. For ‘free text’ answer options please do not include any identifying personal information.

74. Did you previously take part in sport or physical activity as a child (aged 12 to 16 years)?

Scale: Running; Cycling; Swimming; Triathlon; Gymnastics; Weightlifting; Combat sports/Martial Arts; Ball sports; Racket sports; Track and Field; Cross-fit; Dance; Other (please specify); No

75. (If yes to 74), did your sport or activity require you to be lean for either aesthetic or performance reasons, or to make weight?

Scale: Yes/No

76. Do you play representative sport in the military?

Scale: Yes, for my Service; Yes, for my Corps/Region/Branch; Yes, for my Unit; No

77. (If yes to 76) Which sport?

Scale: Running; Cycling; Swimming; Triathlon; Gymnastics; Weightlifting; Combat sports/Martial Arts; Ball sports; Racket sports; Track and Field; Other (please specify)

78. (If yes to 76) How many hours per week do you normally train and / or compete?

Scale: Up to 1 hour; between 1 – 3 hours; Between 3- 5 hours; Between 5 – 7 hours; Between 7 – 8 hours; 8 hours or more

79. How many hours per week do you normally complete physical training as part of your job?

Scale: Up to 1 hour; between 1 – 3 hours; Between 3- 5 hours; Between 5 – 7 hours; Between 7 – 8 hours; 8 hours or more

80. What type of physical training do you currently do as part of your job?

Scale: Running; Cycling; Swimming; Weightlifting; Gym exercises (eg: circuits, cross trainer, gym stair climber); Military specific training (e.g., load carriage, combat physical training); None; Other (please specify)

81. How many hours per week do you normally complete physical training in your personal time?

Scale: Up to 1 hour; 1 – 3 hours; 3- 5 hours; 5 – 7 hours; 7 – 8 hours; 8 hours or more

82. What type of physical training do you do in your personal time?

Scale: Running; Cycling; Swimming; Weightlifting; Gym exercises (eg: circuits, cross trainer, gym stair climber); Team sports; Military specific training (e.g., load carriage, combat physical training); Other (please specify)

83. Has the amount of exercise you do changed since the initial COVID-19 ‘Lockdown’ on 23rd March 2020?

Scale: No; I exercise a lot more now; I exercise a bit more now; I exercise a bit less now; I exercise a lot less now.

84. Have you missed any training in the last year due to injuries?
Scale: No, not at all; Yes, once or twice; Yes, three or four times, Yes, five or more times

85. If yes to 84, how many days were you absent from training or work due to injuries in the last year?
Scale: 1-7 days; 8-14 days; 15-21 days; 22 days or more.

86. If yes to 84, what type of injury did you have?
Scale: Soft tissue injury (muscle, tendon, ligament) – lower body; Soft tissue injury (muscle, tendon, ligament) – upper body; Bone fracture – lower body; Bone stress injury – lower body; Bone fracture – upper body; Bone stress injury – upper body; Dislocation – upper body; Dislocation – lower body; Head/neck injury; Back/spine injury; Hip injury (if none of the above); Ankle/foot injury (if none of the above); Army injury (if none of the above); Other (please specify)

87. Have you missed any training in the last year due to illness?
Scale: No, not at all; Yes, once or twice; Yes, three or four times, Yes, five or more times

88. (If yes to 87) How many days were you absent from training or work due to illness in the last year?
Scale: 1-7 days; 8-14 days; 15-21 days; 22 days or more.

89. (If yes to 87) What type of illness did you have?
Scale: Cold or flu; Diarrhoea, stomach illness or vomiting; Other

90. Do you ever get a gaseous or bloated feeling in your stomach that is not related to your period?
Scale: Yes, several times a day; Yes, several times a week; Yes, once or twice a week or more; Rarely or never.

91. Do you ever get cramps or stomach-ache that is not related to your period?
Scale: Yes, several times a day; Yes, several times a week; Yes, once or twice a week or more; Rarely or never.

92. How often do you have bowel movements on average?
Scale: Several times a day; Once a day; Every second day; Twice a week; Once a week or more rarely.

93. How would you describe your normal stool?
Scale: Normal (soft); Diarrhoea-like (watery); Hard and dry

Section E: About Your Periods (LEAF-Q [94 to 95, 98 to 99, and 101 to 113])
For 'free text' answers, please do not include any information that may identify you.

94. Do you use hormonal contraceptives? (Hormonal contraceptives are defined as, a method of contraception that alters the hormonal profile to prevent pregnancy and/or to control the menstrual cycle. These include, the combined pill, the 'mini' pill (progesterone only), the contraceptive implant or injection, or the Mirena coil).
Scale: Yes; No

95. (If yes to 94) What type of hormonal contraceptive do you currently use?
Scale: Combined Oral Contraceptive Pill (e.g., Microgynon); Progesterone Only Oral Contraceptive Pill/Mini Pill (e.g., Micronor); Contraceptive Implant (e.g., Nexplanon); Contraceptive Injection (e.g., Depo-Provera); Hormonal Intrauterine Device (e.g., Mirena Coil); Vaginal ring; Other (please specify)

96. (If yes to 94) How long have you used your current hormonal contraceptive for?
Scale: Less than 1 year; 1-2 years; 2-5 years; 5 years or more

97. (If yes to 94) Why do you use hormonal contraceptives?
Scale: To prevent pregnancy; Reduce period pains; Reduce bleeding; To regulate the menstrual cycle in relation to performance or work; Other (please specify)

98. (If no to 94) Have you ever used hormonal contraceptives?
Scale: Yes; No

99. (If yes to 98) What type?
Scale: Combined Oral Contraceptive Pill (e.g., Microgynon); Progesterone Only Oral Contraceptive Pill/Mini Pill (e.g., Micronor); Contraceptive Implant (e.g., Nexplanon); Contraceptive Injection (e.g., Depo-Provera); Hormonal Intrauterine Device (e.g., Mirena Coil); Vaginal ring; Other (please specify)

100. (If yes to 98) How long did you use it/them for?
Scale: (short free text)

101. How old were when you had your first period?
Scale: 11 years or younger; 12-14 years; 15 years or older; I do not remember; I have never had a period

102. (if yes to 100) Did your first period come naturally (without medical intervention)?
Scale: Yes; No; I do not remember

103. (If no to 100) What kind of treatment was used to start your periods?
Scale: Hormonal treatment; Weight gain; Reduced volume of exercise; Other

104. (If 'I've never had a period' is not selected for question 100) Do you have normal periods? A normal period is 9 days or less in duration and not so heavy as to soak through personal hygiene products
Scale: Yes; No; Not applicable due to the hormonal contraceptive I currently use

105. (If 'I've never had a period' is not selected for question 100) Are your periods painful (impact on your daily function)?

Scale: Yes; No; Somewhat

106. (If 'I've never had a period' is not selected for question 101) When was your last period?
Scale: 0-4 weeks ago; 1-2 months ago; 3-4 months ago; 5 months ago or more

107. (If 'I've never had a period' is not selected for question 101) Are your periods regular? (every 21 to 34 days)
Scale: Yes; No

108. (If 'I've never had a period' is not selected for question 101) How many days do you normally bleed?
Scale: 1-2 days; 3-4 days; 5-6 days; 7-8 days; 9 days or more

109. (If 'I've never had a period' is not selected for question 101) Have you ever had heavy menstrual bleeding? A good indication that your periods are heavy is if you: are having to change your sanitary products every hour or 2; are passing blood clots larger than 2.5cm (about the size of a 10p coin; are bleeding through your clothes or bedding; need to use 2 types of sanitary products together (for example, tampons and pads).
Scale: Yes; No

110. How many periods have you had during the last year?
Scale: 12 or more; 9-11; 6-8; 3-5; 0-2; None due to the hormonal contraceptive I currently use

111. Have your periods ever stopped for 3 consecutive months or longer (besides pregnancy or because of contraception)?
Scale: No, never; Yes, previously; Yes, now

112. Do your periods change when you increase your exercise intensity, frequency, or duration?
Scale: Yes; No

113. If yes to 112, how?
Scale: I bleed less; I bleed fewer days; My periods stop; I bleed more; I bleed more days

114. Have your periods changed since the initial COVID-19 'Lockdown' on 23rd March 2020?
Scale: Yes; No

115. If yes to 114, how?
Scale: I bleed less; I bleed fewer days; My periods stop; I bleed more; I bleed more days

116. Have you have ever been diagnosed as anaemic or as having low iron?
Scale: Yes; No

Section 6: Bone Health

For the 'free text' answers, please do not include any information that may identify you.

117. Have you ever been diagnosed with a bone stress injury (stress response and/or a stress fracture due to over-exercise and not related to a physical trauma)?

Scale: Yes, between 12 and 18 years old; Yes, during basic training; Yes, during trade training; Yes, during other military training; Yes, during military duty outside of training; Yes, for reasons other than military duty; No, I have never had a stress fracture; Not sure

118. (If yes to 117) How many bone stress injuries have you had?

Scale: 1; 2; 3; 4; 5; More than 5

119. (If yes to 117) How long ago was your bone stress injury?

Scale: Within the last year; 1 – 2 years ago; 2 – 3 years ago; 3 – 4 years ago; 4 – 5 years ago; More than 5 years ago

120. (If yes to 117) Have you ever had more than one bone stress injury in a year?

Scale: Yes; No

121. (If yes to 117) Where was the location of the stress fracture?

Scale: Foot; Shin; Hip/Pelvis; Other (please specify)

122. (If yes to 117) Were you having regular periods at the time of your stress fracture?

Scale: Yes; No; I don't know

123. Have you ever been diagnosed with osteopenia or low bone mineral density?

Scale: Yes; No

124. Do you have a family history of osteoporosis?

Scale: Yes; No; I don't know

Section 7: Anxiety

125. Have you ever had anxiety or depression or anxiety that has required professional and/or medical treatment?
Scale: Yes/No

126. Has your mental health changed since the initial COVID-19 ‘Lockdown’ on 23rd March 2020?
Scale: No; Yes, it is significantly better; Yes, it is slightly better; Yes, it is slightly worse; Yes, it is significantly worse

Over the last 2 weeks, how often have you been bothered by the following problems?

127. Feeling nervous, anxious or on edge
Scale: Not at all; several days; more than half the days; nearly every day

128. Not being able to stop or control worrying
Scale: Not at all; several days; more than half the days; nearly every day

129. Worrying too much about different things
Scale: Not at all; several days; more than half the days; nearly every day

130. Trouble relaxing
Scale: Not at all; several days; more than half the days; nearly every day

131. Being so restless that it is hard to sit still
Scale: Not at all; several days; more than half the days; nearly every day

132. Becoming easily annoyed or irritable
Scale: Not at all; several days; more than half the days; nearly every day

133. Feeling afraid as if something awful might happen
Scale: Not at all; several days; more than half the days; nearly every day

134. If you checked off any problems, how difficult have these problems made it for you to do your work, take care of things at home, or get along with other people?
Scale: Not difficult at all; somewhat difficult; very difficult; extremely difficult; Not applicable

Section 8: Depression

Over the last 2 weeks, how often have you been bothered by any of the following problems?

135. Little interest or please in doing things?
Scale: Not at all; several days; more than half the days; nearly every day

136. Feeling down, depressed, or hopeless?
Scale: Not at all; several days; more than half the days; nearly every day

137. Trouble falling or staying asleep, or sleeping too much?
Scale: Not at all; several days; more than half the days; nearly every day

138. Feeling tired or having little energy
Scale: Not at all; several days; more than half the days; nearly every day

139. Poor appetite or overeating?
Scale: Not at all; several days; more than half the days; nearly every day

140. Feeling bad about yourself – or that you are a failure or have let yourself or your family down?
Scale: Not at all; several days; more than half the days; nearly every day

141. Trouble concentrating on things, such as reading the newspaper or watching television?
Scale: Not at all; several days; more than half the days; nearly every day

142. Moving or speaking so slowly that other people could have noticed? Or the opposite – being too fidgety or restless that you have been moving around a lot more than usual?
Scale: Not at all; several days; more than half the days; nearly every day

143. Thoughts that you would be better off dead, or of hurting yourself in some way?
Scale: Not at all; several days; more than half the days; nearly every day

144. If you checked off any problems, how difficult have these problems made it for you to do your work, take care of things at home, or get along with other people?
Scale: Not difficult at all; somewhat difficult; very difficult; extremely difficult; Not applicable

Section 9: Stress

The questions in this scale ask you about your feelings and thoughts during the **last month**. In each case, you will be asked to indicate how ***often*** you felt or thought a certain way. Although some of the questions are similar, there are differences between them, and you should treat each one as a separate question.

145. In the last month, how often have you been upset because of something that happened unexpectedly?
Scale: Never; almost never; sometimes; fairly often; very often

146. In the last month, how often have you felt that you were unable to control the important things in your life?
Scale: Never; almost never; sometimes; fairly often; very often

147. In the last month, how often have you felt nervous and "stressed"?
Scale: Never; almost never; sometimes; fairly often; very often

148. In the last month, how often have you felt confident about your ability to handle your personal problems?
Scale: Never; almost never; sometimes; fairly often; very often

149. In the last month, how often have you felt that things were going your way?
Scale: Never; almost never; sometimes; fairly often; very often

150. In the last month, how often have you found that you could not cope with all the things you had to do?
Scale: Never; almost never; sometimes; fairly often; very often

151. In the last month, how often have you been able to control irritations in your life?
Scale: Never; almost never; sometimes; fairly often; very often

152. In the last month, how often have you felt that you were on top of things?
Scale: Never; almost never; sometimes; fairly often; very often

153. In the last month, how often have you been angered because of things that were outside of your control?
Scale: Never; almost never; sometimes; fairly often; very often

154. In the last month, how often have you felt difficulties were piling up so high that you could not overcome them?
Scale: Never; almost never; sometimes; fairly often; very often

Section 10: Resilience

The questions in this scale ask you about your resilience. Please answer the following statements as they apply to you over the **last month**. If a particular situation has not occurred recently, answer according to how you would have think you would have felt.

155. I am able to adapt when changes occur.
Scale: Not at all true; rarely true; sometimes true; often true; true nearly all the time

156. I can deal with whatever comes my way.
Scale: Not at all true; rarely true; sometimes true; often true; true nearly all the time

157. I try to see the humorous side of things when I am faced with problems.
Scale: Not at all true; rarely true; sometimes true; often true; true nearly all the time

158. Having to cope with stress can make me stronger.
Scale: Not at all true; rarely true; sometimes true; often true; true nearly all the time

159. I tend to bounce back after illness, injury, or other hardships.
Scale: Not at all true; rarely true; sometimes true; often true; true nearly all the time

160. I believe I can achieve my goals, even if there are obstacles.
Scale: Not at all true; rarely true; sometimes true; often true; true nearly all the time

161. Under pressure, I stay focussed and think clearly.
Scale: Not at all true; rarely true; sometimes true; often true; true nearly all the time

162. I am not easily discouraged by failure.
Scale: Not at all true; rarely true; sometimes true; often true; true nearly all the time

163. I think of myself as a strong person when dealing with life’s challenges and difficulties.
Scale: Not at all true; rarely true; sometimes true; often true; true nearly all the time

164. I am able to handle unpleasant or painful feelings like sadness, fear, and anger.
Scale: Not at all true; rarely true; sometimes true; often true; true nearly all the time

Section 11: Physical Performance

Army

165. When was your last physical performance test?
Scale: Less than 3 months ago; 3 to 6 months ago; 6 to 12 months ago; More than 12 months ago

166. What was your latest 2 km run time or multi-stage fitness test result?
Scale: Short free text

167. What was your latest broad jump score?
Scale: Short free text

168. What was your latest medicine ball throw score?
Scale: Short free text

169. What was your latest deadlift (hexbar lift) score?
Scale: Short free text

170. What was your latest 100 m shuttle sprint score?
Scale: Short free text

171. What was your latest pull-up score?
Scale: Short free text

Navy

172. When was your last physical performance test?
Scale: Less than 3 months ago; 3 to 6 months ago; 6 to 12 months ago; More than 12 months ago

173. What was your latest 2.4 km run time, multi-stage fitness test result, or Rockport walk score?
Scale: Short free text

174. What was your latest strength test score?
Scale: Short free text

Royal Air Force

175. When was your last physical performance test?
Scale: Less than 3 months ago; 3 to 6 months ago; 6 to 12 months ago; More than 12 months ago

176. What was your latest multi-stage fitness test or Rockport Walk test result?
Scale: Short free text

177. What was your latest press-up score?
Scale: Short free text

178. What was your latest sit-up score?
Scale: Short free text
